# Mission in Sukusuku Cohort, Mie: Focusing on the Feasibility and Validity of Methods for Enrolling and Retaining Participants

**DOI:** 10.2188/jea.JE20090165

**Published:** 2010-03-05

**Authors:** Noriko Yamakawa, Haruka Koike, Noriko Ohtani, Motoki Bonno, Shigeki Tanaka, Masaru Ido, Yoshihiro Komada, Masatoshi Kawai, Hatsumi Yamamoto

**Affiliations:** 1 Clinical Research Institute, Mie-chuo Medical Center, National Hospital Organization, Tsu, Japan; 2 Department of Neonatal Science, Institute of Molecular and Experimental Medicine, Mie University Graduate School of Medicine, Tsu, Japan; 3 Pediatrics and developmental Science, Institute of Molecular and Experimental Medicine, Mie University Graduate School of Medicine, Tsu, Japan; 4 Center for the study of child development, Institute for Education, Mukogawa Women’s University, Nishinomiya, Hyogo, Japan

**Keywords:** Japan Children’s Study, Sukusuku Cohort Mie, feasibility, validity, enrollment, retention rate

## Abstract

**Background:**

We investigated the feasibility and validity of and systematized the methods used to enroll and retain participants requiring long-term interdisciplinary collaborations. We carried out this study in the Sukusuku cohort, Mie (SCM), as one of the regional research site of Japan Children’s Study (JCS).

**Methods:**

A total of 467 families who were screened between December 1, 2004 and December 31, 2005, in the Mie-chuo Medical Center and 2 other hospitals; these families were deemed eligible for the study. Of these, a total of 185 families (39.6%) participated in the 4-month observation. Of these families, 5 dropped out at month 9 of the observation; 9, at month 18; 17, at month 30; and 5, at month 42. The retention rates at 9, 18, 30, and 42 months of observation were 97.3%, 92.4%, 83.2%, and 80.5%, respectively. Reinstatement to a previous job was the most common reason for dropouts.

**Results:**

We observed that informative consultation notes during observation were beneficial for the retention of participants, and these notes also helped in improving communication between the study subjects and the evaluators during subsequent visits.

**Conclusions:**

In this study, we did not perform the standard checks for child development alone but also investigated the motivating influence of research partnerships with participants. Further, these visits help maintain the motivation levels of the participants and encourage them to contribute for social causes. The results present integration models that can be applied in future relevant longitudinal cohort studies in Japan.

## INTRODUCTION

We aimed to identify the factors influencing cognitive-behavioral development of children in Japan. To this end, a cohort study named Japan Children’s Study (JCS) was initiated by the Research Institute of Science and Technology for Society, Japan Science and Technology Agency (RISTEX/JST) in 2004. Therefore, we cooperated with the Health and Welfare departments and the board of education of the local government in the Sukusuku cohort in Mie Prefecture.

Initially, the Sukusuku cohort, Mie (SCM) was organized to promote JCS; this project involved the President, Mie University; the Dean of the School of Medicine, Mie University; the local government of Mie Prefecture, Owase City; and the pediatric and Obstetrics/Gynecology societies in Mie Prefecture. The study design in SCM was arranged in a progressive discussion by all the abovementioned parties. Symposia on the subject of “Pursuit of child development: Responsibilities in the 21^st^ century” were held to educate the population of Tsu and Owase cities and to raise awareness about the importance of the study.^1^

Here, we primarily aimed to examine the feasibility and validity of the methods by which participants that need to be observed for a long term can be enrolled and retained.^2^ Many studies have investigated the factors influencing the retention rate of participants to identify developing diseases^3^^–^^8^; however, in our study, we investigated the strategies and features employed to maintain the retention rate of participants in a prospective long-term cohort study on healthy Japanese children.

## METHODS

### Sample enrollment

The sampling procedure for this study was designed to include families with healthy infants. Participants were recruited from Mie-chuo Medical Center, Tsu City; Saint Rose Clinic, Tsu City; and Owase General Hospital, Owase City. All babies born between December 1, 2004, and December 31, 2005, in these medical centers were screened during their postpartum period. Participants were excluded from the study if: (1) the family planned to move from the area within a year; (2) the infant was hospitalized in the neonatal intensive care unit (NICU) for over one week; (3) the mother refused to participate in the study; (4) the mother lived outside Mie prefecture; (5) the mother was a foreigner; (6) the infant aged younger than 4.0 months and older than 4.5 months at the first observation scheduled at 4 months of infant age.

### Hospital screening in Mie-chuo Medical Center

The project outlines were explained to the participants in a video presentation during antenatal childbirth classes in Mie-chuo Medical Center. Babies born between December 1, 2004, and December 31, 2005, were screened at Mie-chuo Medical Center. The eligible participants that visited the hospital screenings were used to generate a list of eligible families for the 2-week follow-up examination of the infants. During these screenings, the participants were provided complete instructions regarding the research program by a pediatrician or a clinical research coordinator (CRC), and informed consents were obtained from the parents. Families were officially enrolled in the study if they successfully completed written informed consent at the 1-month medical examination.

### Screening in Saint Rose Clinic, Tsu City

Infants born between May and November 2005 were screened at Saint Rose Clinic, Tsu City. At the time of the one-month medical examination, a pediatrician or CRC explained the detailed study plans to the infants’ mothers. Those who consented to participate in this study submitted their consents at the same visit; those who consulted with families mailed their consent forms later in self-addressed envelopes. One week after the 1-month visit, we telephoned the mothers who consented to the study at that visit to reconfirm their consent, and they were given an appointment at a suitable time for the first visit.

### Screening in Owase General Hospital

Families that visited Owase General Hospital for the birth, medical examination, or vaccination of their infants between April 2005 and December 2005 were informed of the study by a pediatrician of this hospital. The subjects also provided informed consents to participate in this study at the 1-month medical examination or by mail. As mentioned above, one week after the 1-month visit, we telephoned the families who consented to the study at that visit to reconfirm their consents, and they were given an appointment at a suitable time for the first visit.

All the consent forms and observation schedules were preserved and maintained by the SCM secretariat in Mie Chuo Medical Center.

### Enrollment and retention strategies

Certain strategies were adopted to retain the participants throughout the longitudinal study (Table 1). The CRC sent a questionnaire and reminders to the participants 2 weeks before the observation. The visits were fully flexible, scheduled according to the subject’s convenience, and there was no waiting time. The patient had the flexibility to reschedule the appointment if any of the visits were cancelled or missed. After the observation, all the children were given a small gift and a postcard with their own photograph. If the mothers requested consultation, a qualified pediatrician provided free consultation on childcare and vaccination and medical examination and explained the results to the mothers after the observation. The mothers also received free parking tickets after each observation. Further, all the families received birthday greeting cards.

**Table 1.** Our strategies to retain participants

1.	Flexibility in scheduling of visits
2.	Daycare for the infant’s siblings during the observation
3.	Observing the participants during the consultation and recording this information
4.	Providing personalized attention to the participant’s family
5.	Timely consultation for child rearing by a well-trained pediatrician
6.	“Good for children” aspect of the study staff to motivate participants
7.	Preparation of a home-like atmosphere in the observation room
8.	Premium gifts to the parents to encourage child development
9.	Regular approach with a written invitation before the observation and a letter of thanks after the visit
10.	Timely feedback of the results to participants via newsletters and forums

### Statistical analysis

Statistical analyses to compare the participants’ level of motivation across groups were performed by Student’s *t* test or chi-square test, and a value of *P* < 0.05 was considered statistically significant.

## RESULTS

### Study participants

Of the 467 families that were deemed eligible for participation in this study, 199 families (42.6%) consented to participate. Of these, 113 of 247 families (45.7%) were in Mie-chuo medical center, 48 of 132 (36.4%) in Saint Rose Clinic, 24 of 74 (32.4%) in Owase General Hospital, and 14 of 14 (100%) who were notified by word of mouth consented to participate in this study (Table 2). Of these 199 families, 14 dropped out at the fourth month of observation. The 185 families (39.6%) that continued beyond the fourth month of observation were analyzed in this study.

**Table 2.** Number of families enrolled in this study

Number of families	Mie-chuo medical center		A neighboring clinic	Owase general hospital	By word of mouth	Total

	Initiation of study					

	Before	After				
Eligible	85	162	132	74	14	467

Consented	28	85	48	24	14	199
	32.9%	52.5%	36.4%	32.4%	100.0%	42.6%
	45.7%					

Four month	26	82	40	23	14	185
observation	30.6%	50.6%	30.3%	31.1%	100.0%	39.6%
	43.7%					

### Retention rate

Table 3 represents the retention rate at month 4, 9, 18, 30, and 42 of observation. Of the 185 families that continued in the study beyond the fourth month, 5 families dropped out at month 9; 9 families, at month 18; 17 families, at month 30; and 5 families, at month 42. The retention rates at months 9, 18, 30, and 42 were 97.3%, 92.4%, 83.2%, and 80.5%, respectively. The retention rates at 42 months were 83.3% (90 families) in Mie-chuo Medical Center, 82.5% (33 families) in Saint Rose Clinic, 60.9% (14 families) in Owase General Hospital, and 85.7% (12 families) in the subjects recruited by word of mouth, respectively (data not shown).

**Table 3.** Retention rate

	Number of participants	Out of follow-up	Retention rate
4 months	185		100%
9 months	180	5	97.30%
18 months	171	9	92.40%
30 months	154	17	83.20%
42 months	149	5	80.50%

### Motivation to participate

The responses to the questionnaire revealed that the observation of child growth by a pediatric specialist was the strongest motivator of participation in this study. Child rearing consultation was also one of the main motivators for attending the initial visits. From the second visit onwards, the realization that participation in this study is a way of contributing to a social cause and monitoring of the child’s growth were also incentives. Participants anticipating child-rearing support participated in the study consistently because of the opportunity to observe their child’s development from a social viewpoint. In the questionnaire, participants were queried about future observations and more participants responded “really looking forward to participate in future observations” as compared to “would like to participate in future observations if time permits,” indicating that the participants were keen on attending future visits (*P* < 0.05, data not shown). Thus, we consider that eager participation rather than mere voluntary participation is a key to retain participants (*P* < 0.05, data not shown).

### Reason for dropouts

As shown in Table 4, reinstatement to former jobs was the most common reason for dropouts from this study. Of the 36 dropouts, 12 returned to their former jobs. Nine of the mothers did not revert. Seven subjects were unwell and consequently were unable to continue participating in the study. Four participants dropped out of the study because they were pregnant. Three subjects moved to another city and one child started kindergarten. The participants’ employment rates gradually increased from 10.8% at 4 months to 14.4% at 9 months, 36.8% at 18 months, and 49.4% at 42 months. According to the labor force survey annual report, the employment rate of mothers with children up to 3 years of age in Japan was 34.1%, and the average employment rate of the participants in our study from 9 to 30 months of observation was similar at 32.7%.

**Table 4.** The reason for dropping out of follow-up observation

	9 months	18 months	30 months	42 months	Total
Reinstatement to former job	1	4	6	1	12
Lost to follow-up	2	2	2	3	9
Illness	2	1	4		7
Pregnant		1	2	1	4
Moved to another city		1	2		3
Children started kindergarten			1		1

Total	5	9	17	5	36

## DISCUSSION

Longitudinal cohort studies on children from birth through childhood provide a rare opportunity to observe and monitor the developmental process in children. Many factors influence the retention rate of participants in longitudinal cohort studies. In our study, we examined the feasibility and validity of the methods used to enroll and retain participants because a high retention rate plays a crucial role in preventing potential bias due to missing data, which threatens the internal validities of the study and reduces the reliability of the outcome.

In previous cohort studies, free checkups for the mothers and babies resulted in a 31% retention rate in a 7-year study^8^ and a $100 incentive per subject for the successful completion of a 4-year study resulted in a retention rate of 66%.^9^ However, retention rates of 92% in a 5-year study,^10^ 94% in a 3-year study,^11^ and 81% in a 9-year study^12^ were successfully achieved without any incentives to the mother. These high retention rates have been reported to be due to the quality of the health care system, communication between the healthcare professionals and participants, and publicity of the survey results.^10^^–^^12^ Moreover, the National Longitudinal Survey of Children reported a 66% retention rate with 2-hour interviews at the mother’s home,^13^ whereas a Queensland study, which had three 100-question interviews, had retention rates of 99%, 87%, and 81% over the 3 phases of the study.^14^ Previous studies have reported that the duration of the observation or interview did not have any association with the retention rate; whereas change in telephone number or moving residence was reported as one of the most important reasons for dropout of the participants.^15^^,^^16^

According to this child cohort study, the following factors may influence the retention rate of the participants: (1) flexibility in scheduling of visits, (2) knowledgeable, well-trained, motivated, and persuasive study staff that establishes a good rapport with the participants and are persistent in obtaining responses, (3) timely feedback, including quick responses to the participant’s questions and rapid results of the medical tests taken, (4) personalized attention to the study participants, (5) personal visits to the homes of the participants, (6) information to the participants that they can contribute to a social cause, which is a motivating factor for participation in the study.

On this account, we changed the period of recruitment from the puerperal period to the medical examination at the second or fourth week, since the mothers were in good health at the second week than during the puerperal period. At this time, many mothers could be efficiently explained the details of the study, resulted in their better understanding and a higher enrollment rate. Further, since it is difficult to attend to all the mothers in a group, we explained the details of the study individually to each mother while requesting their participation in the study.

**Figure 1.**
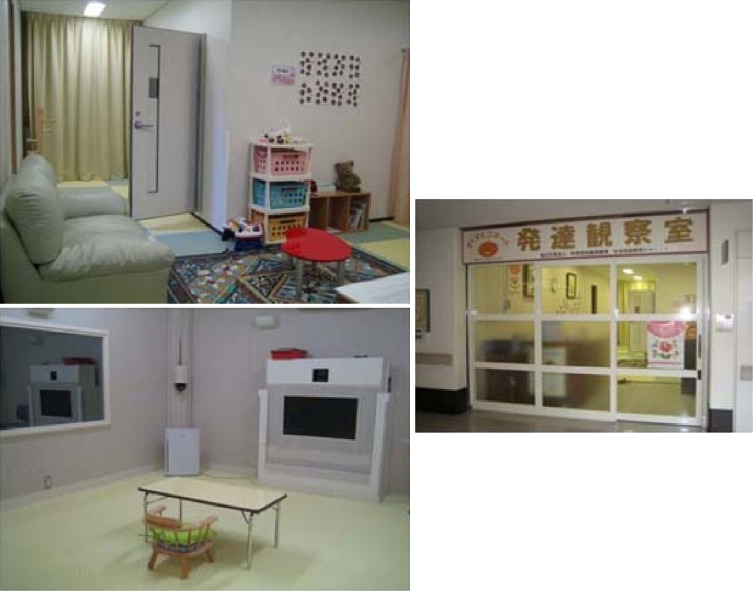
An observation room with homelike atmosphere and music.

**Figure 2.**
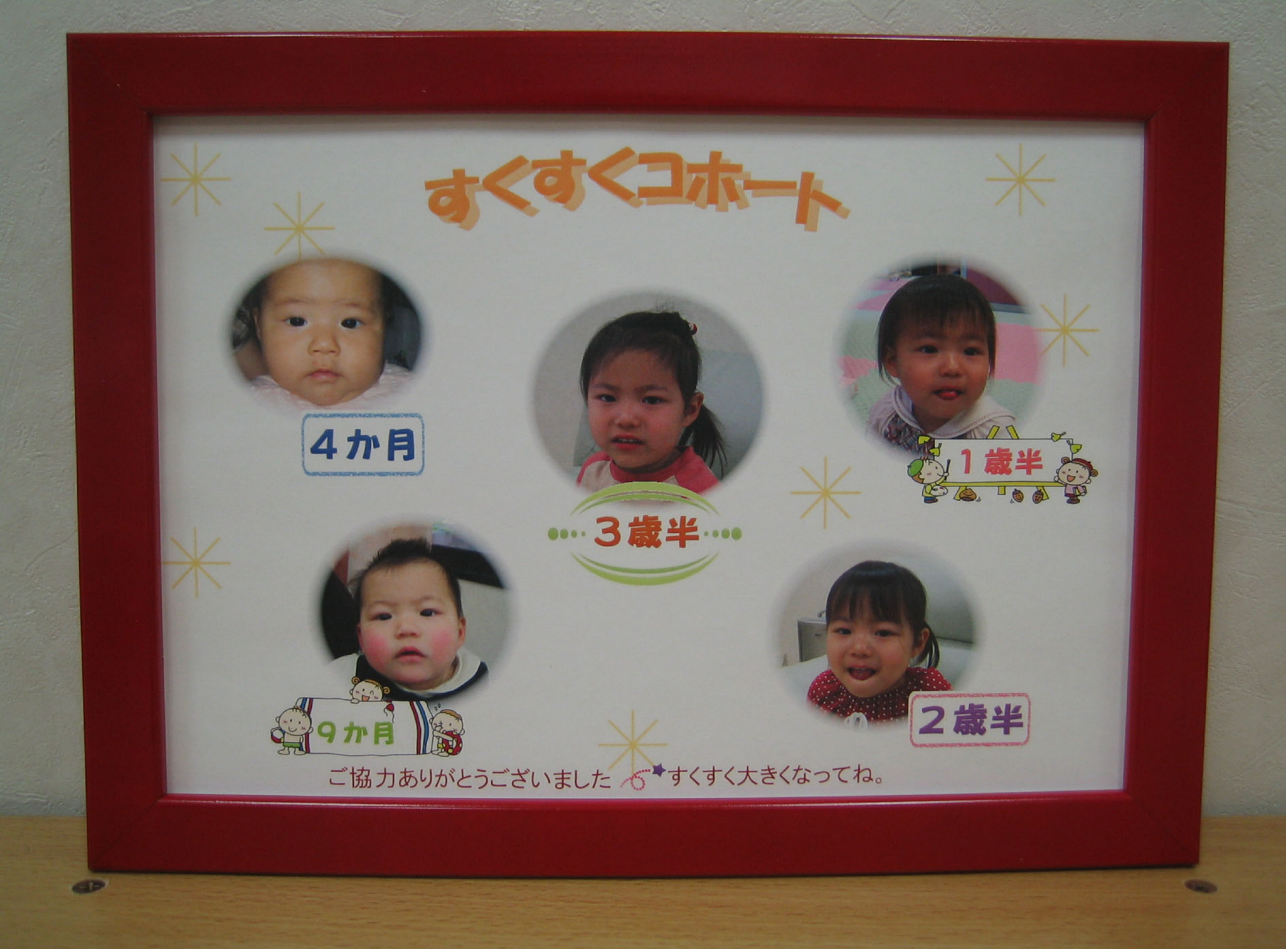
A photo collage portraying a child’s development throughout the study period. Individual photographs were taken at each observation. The collage was framed and presented to the family at the end of month 42 of observation. (This girl’s parents agreed to insert the photograph on the paper.)

However, an important aim of this study was to enroll not only families with a strong interest in child development but also those with less interest in child development. Therefore, we adopted strategies such as a full illustration of our observations, introduction of medical observation or vaccinations, a regular approach with a written invitation sent 2 weeks prior to the appointment, a free parking ticket, preparation of a homelike atmosphere with ambient music in the observation room (Figure 1), and flexible schedules for the observation with no waiting time. Further, at the end of every observation, the participants received small gifts such as book coupons, toys, an item that can be used for child care, and the child’s photograph for their attendance. In particular, the children’s photographs were taken at each observation and edited into 1 photo collage to portray the child’s development throughout the period of the study (Figure 2). This photograph was framed and presented to the family at the end of the 42 months of observation to motivate continuous participation. Moreover, all participants received a letter of thanks after each visit, and birthday, Christmas, and New Year’s cards and newsletters were sent to all participants to increase their awareness (Table 1).

The results of the motivational analysis revealed that the subject who participated in the study with pleasure had the strongest motivation. These results suggested that eager participation rather than mere voluntary participation played an important role in retaining participants in a study.

Reinstatement to the former job was the most common reason for a mother to drop out of the study. Further, 5 of the mothers who participated in the study did not revert. Although reinstatement was the primary reason for dropping out of the study, the employment rate of the participants remained the same as that of the general population at 30 months of the study. Work and physical unfitness of the mother were also considered important reasons for missing follow-up visits in our study. In order to retain participants over a long period, it is important to record 2 or more contact addresses and to send mails to obtain information in case the subject shifts to another location.

The retention rates in our study were 97.3%, 91.8%, 83.2%, and 80.5% at 9, 18, 30, and 42 months of observation. However, it is difficult to ascertain the specific reason for the high retention rate in our study, although we consider that informative correspondents, efficient communication, informing the participants about the schedule over the telephone, good communication of the study intents, good relationship between the participants and the physician, face-to-face interviews, and community involvement may have influenced the retention rate. Further, data accumulation and support with respect to raising the child were helpful in retaining participants. Moreover, informative consultation notes of the participants during observation were very useful for establishing good communication for the subsequent visits. This note contained important information about the families, such as the arrival time, person accompanying the child, tests that they were unable to perform during the observation, appearance, sleepiness, hunger, attitude towards the study, physical condition, and method of providing childcare.

Our results suggest that successful retention rates in a birth cohort study highly depended on good communication and formation of a partnership between the participants and the physician, as previously reported.^11^ Furthermore, we did not perform the standard checks for child development alone but also investigated the motivating influence of research partnerships with participants. Further, these visits help maintain the motivation levels of the participants and encourage them to contribute for social causes.

## CONCLUSION

We investigated the feasibility and validity of the methods used to enroll and retain participants requiring long-term interdisciplinary collaborations in this SCM study. Maximum sample size and minimum dropout rate are required to perform adequate statistical analysis. In this study, we observed that good communication and forming partnerships with the participants were extremely useful strategies for maintaining high retention rates. These results will be a good model for future relevant longitudinal cohort studies in Japan.
